# Reverse-transcriptase real-time PCR in the diagnostic strategy for invasive infections caused by *Aspergillus fumigatus*

**DOI:** 10.1128/jcm.00791-24

**Published:** 2024-10-24

**Authors:** Charles Gibert, Pauline Tirard-Collet, Charline Miossec, Damien Dupont, Florence Persat, Martine Wallon, Florence Ader, Gilles Devouassoux, Sophie Ducastelle, Hélène Labussière-Wallet, Sylvie Paulus, Céline Guichon, Anne-Claire Lukaszewicz, Jean-Christophe Richard, Florent Wallet, Alexandre Alanio, Meja Rabodonirina, Jean Menotti

**Affiliations:** 1 Hospices Civils de Lyon, Laboratoire de Parasitologie et Mycologie Médicale, Institut des Agents Infectieux, Lyon, France; 2 Université Claude Bernard Lyon 1, Villeurbanne, France; 3 Hospices Civils de Lyon, Services de Maladies Infectieuses et Tropicales, Hôpital de la Croix-Rousse, Lyon, France; 4 Hospices Civils de Lyon, Service de Pneumologie, Hôpital de la Croix-Rousse, Lyon, France; 5 Hospices Civils de Lyon, Service d’Hématologie Clinique, Hôpital Lyon Sud, Pierre-Bénite, France; 6 Hospices Civils de Lyon, Service d'Anesthésie-Réanimation, Hôpital Louis Pradel, Bron, France; 7 Hospices Civils de Lyon, Service d'Anesthésie-Réanimation, Hôpital de la Croix-Rousse, Lyon, France; 8 Hospices Civils de Lyon, Service d'Anesthésie-Réanimation, Hôpital Edouard Herriot, Lyon, France; 9 Hospices Civils de Lyon, Service de Médecine intensive, Hôpital de la Croix-Rousse, Lyon, France; 10 Hospices Civils de Lyon, Service d'Anesthésie-Réanimation-Médecine intensive, Hôpital Lyon Sud, Lyon, France; 11 Assistance Publique-Hôpitaux de Paris, Laboratoire de Parasitologie-Mycologie, Hôpital St-Louis, Paris, France; 12 Institut Pasteur, Université Paris-Cité, Centre National de Référence Mycoses Invasives et Antifongiques, Groupe de Recherche Mycologie Translationnelle, Département de Mycologie, Paris, France; University of Utah, Salt Lake City, Utah, USA

**Keywords:** *Aspergillus fumigatus*, molecular diagnosis, reverse transcriptase PCR, invasive aspergillosis, fungal infection

## Abstract

The aim was to develop an RT-qPCR targeting *Aspergillus fumigatus* and compare its performance to that of *Aspergillus fumigatus* qPCR for the diagnosis of invasive aspergillosis (IA). Samples from patients of the Lyon University hospitals for whom a suspicion of IA led to the realization of an *Aspergillus fumigatus* qPCR molecular diagnostic test over a 2-year period were included. The patients were classified according to the European Organization for Research and Treatment of Cancer/Mycoses Study Group (EORTC-MSGERC) criteria for suspected IA; RT-qPCR and qPCR assays were performed on all included samples. The sensitivities and specificities of RT-qPCR and qPCR were calculated and compared using the results of the EORTC-MSGERC classification as reference. The cycle threshold (Ct) results were compared according to IA classification and sample type. Among the 193 samples analyzed, 91 were classified as IA excluded, 46 as possible IA, 53 as probable IA, and 3 as proven IA. For all sample types, RT-qPCR was significantly more sensitive than qPCR for all IA classifications with an additional 17/102 samples detected (*P*-value < 0.01). For plasma samples, sensitivity was significantly higher and specificity significantly lower using RT-qPCR for all IA classifications (*P*-value < 0.001). The mean Ct obtained with RT-qPCR were significantly lower than those obtained with qPCR for all IA classifications and all sample types (*P*-value < 0.001 and *P*-value < 0.0001, respectively). RT-qPCR presents a higher sensitivity than qPCR for the diagnosis of IA due to *Aspergillus fumigatus*, particularly in samples with an intrinsically low fungal load.

**IMPORTANCE:**

*Aspergillus fumigatus* belongs to the critical priority group of the World Health Organization fungal priority pathogens list. Invasive aspergillosis (IA) is a life-threatening infection with poor prognosis and challenging diagnosis. PCR has been integrated into the 2020 European Organization for Research and Treatment of Cancer/Mycoses Study Group consensus definitions for IA diagnosis. However, due to frequent low fungal burdens, its sensitivity needs to be improved. This work presents an innovative method for detecting total nucleic acids, corresponding to both ribosomal RNA and DNA, that enables IA diagnosis with greater sensitivity than conventional techniques, especially in non-invasive samples such as blood, enhancing the monitoring of this infection in high-risk patients.

## INTRODUCTION

*Aspergillus* is a filamentous fungus found ubiquitously in the environment, mainly in soil, decomposing matter, and all types of dust (1). In human pathology, the incidence of aspergillosis is increasing in association with the growing proportion of immunocompromised patients (2–4). Invasive pulmonary aspergillosis (IPA), mostly due to *Aspergillus fumigatus*, is the most frequent type of invasive aspergillosis (IA) and mainly affects patients with hematological malignancy, hematopoietic stem cell transplant, or solid organ transplant undergoing immunosuppressive therapy, as well as patients receiving chemotherapy (5, 6). As clinical diagnosis alone is not sufficient, and the combination of radiological and mycological tools is not perfectly sensitive and specific, the European Organisation for Research and Treatment of Cancer and Mycoses Study Group Education and Research Consortium (EORTC-MSGERC) has established three categories of invasive infection (“Possible,” “Probable,” and “Proven”) in order to classify the diagnostic certainty; the categorization of a patient suspected of having IA is based on clinical and radiological criteria, host factors, and mycological criteria (7). In 2019, the SARS-CoV-2 virus emerged, and subsequently, patients with severe forms of COVID-19 exhibited aspergillosis with similar forms to IA but without complying with the EORTC-MSGERC host factors. For these patients, the COVID-19-associated pulmonary aspergillosis (CAPA) was defined using the Koehler criteria for stratification of diagnostic certainty, in a similar manner to that of IA (8–11).

Mycological criteria play a key role in classifying patients. To assess the patient’s suitability to the mycological criteria, three tools are available. Conventional mycological culture, which can be used on any type of sample, in combination with proteomic techniques such as matrix-assisted laser desorption mass spectrometry (MALDI-TOF MS) enables the identification of fungal species, as well as antifungal susceptibility testing (AST) if required (12). Another approach is based on the detection of the galactomannan (GM) antigen, enabling the detection of the pathogen in blood or bronchoalveolar fluid, mainly via enzyme immunoassay (EIA) or lateral flow assay (LFA) techniques (13, 14). The latest tool is based on molecular biology, which includes various techniques, most notably real-time polymerization chain reaction (qPCR) (15–18). The latter has the advantage of being able to detect small quantities of fungal genetic material and can be used on all samples (19, 20). For invasive pulmonary aspergillosis, only molecular tests in blood samples (whole blood, plasma, or serum) or BAL samples can comply with EORTC-MSGERC mycological criteria.

More recently, reverse transcriptase PCR (RT-PCR) and its real-time version (RT-qPCR) have been developed to amplify ribonucleic acid (RNA) fragments in addition to desoxyribonucleic acid (DNA) fragments of several fungal pathogens, increasing the sensitivity of the techniques through the detection of the total nucleic acid (TNA) (21–23). However, to our knowledge, RT-qPCR techniques for the diagnosis of IA in humans have not been published yet (24, 25).

The aim of the present study was, therefore, to develop an RT-qPCR targeting *Aspergillus fumigatus* and compare its performance to that of *Aspergillus fumigatus* qPCR for the diagnosis of IA.

## MATERIALS AND METHODS

### Study design

The study was carried out at the Hospices Civils de Lyon, France, a tertiary care university hospital with a large capacity for enrolling patients suffering from onco-hematological diseases including hematopoietic stem cell transplant recipients and patients in a state of chronic immunosuppression.

All samples from patients for whom a suspicion of IA due to *Aspergillus fumigatus* led to the prescription and subsequent realization of an *Aspergillus fumigatus* qPCR molecular diagnostic test, between 1 January 2021 and 31 December 2022, were eligible for inclusion. No restriction was placed on the clinical department from which the sample originated, and no clinical data were used for the inclusion. Samples that were still available in the storage biobanks were then included for inclusion.

The clinical, radiological, and mycological data as well as the host factors from the included patient samples were retrieved from medical records to classify them according to the EORTC-MSGERC criteria for suspected IA. PCR results were not used to define the EORTC-MSGERC classification of patients. When these criteria could not allow the classification into possible, probable, or proven categories, the sample was classified as “IA excluded.” Following the classification, sinus samples not corresponding to invasive forms of aspergillosis as well as all samples referring to CAPA were excluded from the analysis.

### Molecular diagnosis

An RT-qPCR assay and a qPCR assay were performed on all included samples, both targeting a 67 bp DNA fragment specific to the multicopy gene encoding *A. fumigatus* 28S rRNA, as previously described (26). TNA extraction from respiratory samples and cerebrospinal fluid (CSF) was performed using the InGenius system (Elitech Group, Puteaux, France) from 1.5 mL of sample for broncho-alveolar liquid (BAL), 500 µL for bronchial and endotracheal aspirates and sputum, and 100 µL of CSF, after a first ultrasound sonication step. For plasma, extraction was performed on a MagNA Pure 24 system (Roche Diagnostics, Basel, Switzerland) from 1 mL of plasma. Biopsies were extracted on a MagNAPure Compact Instrument (Roche Diagnostics). For all sample types, the same nucleic acid extract was used for both RT-qPCR and qPCR analyses.

To perform RT-qPCR, a volume of 9 µL of nucleic acid was added to 16 µL of PCR mix containing SuperScript III Platinum One-Step qRT-PCR (ThermoFisher, Waltham, MA, USA), 0.4 µM each of primers AF28S-F (CTC GGA ATG TAT CAC CTC TCG G) and AF28S-R (TCC TCG GTC CAG GCA GG), 0.2 µM *Aspergillus fumigatus* 28S probe (FAM-TGT CTT ATA GCC GAG GGT GCA ATG CG-BHQ1), and the Simplexa internal control with its primers and probe (Focus Diagnostics, Cypress, CA, USA). For qPCR, a volume of 9 µL of TNA was added to 16 µL of PCR mix containing TaqMan Universal Master Mix II with UNG (ThermoFisher), 0.4 µM each of primers AF28S-F and AF28S-R, 0.2 µM of *Aspergillus fumigatus* 28S probe, and the Simplexa internal control with its primers and probe (Focus Diagnostics), as previously described (26, 27). A calibration curve was previously established using a progressive dilution range from 10^6^ to 10 spores/mL of *Aspergillus fumigatus*. In each run, a positive control concentrated with 10^2^ spores/mL was used as amplification control.

Amplification was performed on a QuantStudio 5 thermal cycler (ThermoFisher) by incubating for 10 min at 95°C, followed by 45 cycles consisting of 15 s of denaturation at 95°C, followed by 60 s of hybridization and elongation at 60°C. An additional 15 min RT step was performed at the start of the reaction for the RT-qPCR. PCR results were defined as positive when an amplification was detected before 45 cycles.

### Other laboratory analysis

Each mycological culture was inoculated onto CAN2 agar (BioMérieux, Marcy-l'Étoile, France) and a Sabouraud agar tube (BioMérieux) for an incubation time that varied according to the sample type. Strain identification was carried out by MALDI-TOF MS using the Vitek MS analyzer (BioMérieux) with the complementary use of the Vitek 3.0 and Mass Spectrometry Identification (MSI) databases, enabling fungal species to be identified with over 99.9% certainty (28). The detection of *Aspergillus fumigatus* galactomannan (GM) antigens was carried out using sandwich enzyme-linked immunosorbent assays (Platelia; BioRad, Marnes-la-Coquette, France) either on BAL or on serum.

### Statistical analysis

The sensitivities and specificities of the RT-qPCR and qPCR were calculated and compared using the results obtained from the EORTC-MSGERC classification as a reference. The sensitivities and specificities were established for different modalities of the EORTC-MSGERC classification results: for all classifications (possible, probable, and proven), for probable and proven IA together, and for probable IA alone. The sensitivities and specificities were also calculated for different sample types: for all samples, for plasma samples only, and for all respiratory samples (bronchial aspirate, endotracheal aspirate, sputum, BAL). All comparisons were made using one-sided Chi^2^ tests. Quantification of the positive, negative, and overall agreement, as well as Kappa coefficient between RT-qPCR and qPCR, was assessed for all classifications and all sample types.

Second, the differences in mean cycle threshold (ΔCt) between qPCR and RT-qPCR were calculated. The ΔCt were established for different modalities of the classification criteria: IA excluded, all classifications (possible, probable, and proven), probable and proven IA together, and probable IA alone. The ΔCt were also established according to different sample types: for all samples, for plasma samples only, and for all respiratory samples. In case of a negative result on either the RT-qPCR or qPCR, a Ct of 45 was considered for the negative one. The ΔCt between qPCR and RT-qPCR were analyzed using two-sided paired *t*-tests. For all tests, a *P*-value of less than 0.05 was considered statistically significant. All statistical analyses were performed using Prism 10.0 software (GraphPad Software, Boston, MA, USA).

## RESULTS

### Participants

Overall, 232 samples from 182 patients were included in the study. After examination of host and clinical criteria, 193 samples were part of a diagnosis of IA, while 26 samples were related to CAPA and 13 related to non-invasive forms of *Aspergillus* sinusitis and were excluded from the analysis. Based on the EORTC-MSGERC criteria, 91 were classified as IA excluded, 46 as possible IA, 53 as probable IA, and 3 as proven IA. The distribution of samples was as follows: 56 plasma samples, 109 respiratory samples, 10 sinus biopsies, 8 CSF, 2 bone samples, and 8 other biopsies (Table 1).

**TABLE 1:** Demographic, clinical, and mycological characteristics of patients and classification of invasive aspergillosis according to consensus definitions from EORTC-MSGERC for the 193 samples considered for analysis*^a^*^,^*^b^*^,^*^c^*

	IA excluded	Possible IA	Probable IA	Proven IA	Total
Samples	91 (47.1)	46 (23.8)	53 (27.5)	3 (1.6)	193
Plasma	27 (29.7)	21(45.7)	7 (13.2)	1 (33.3)	56 (29.0)
Respiratory samples	40 (43.9)	25 (54.3)	43 (81.1)	1 (33.3)	109 (56.5)
Bronchoalveolar liquid	35 (87.5)	20 (80)	29 (16.9)	0	84 (77.1)
Bronchial aspirate	1 (2.5)	2 (8.0)	5 (17.2)	1 (100.0)	9 (8.2)
Endotracheal aspirate	0	1 (4.0)	5 (17.2)	0	6 (5.5)
Sputum	4 (10.0)	2 (8.0)	4 (7.5)	0	10 (9.2)
Sinus biopsies	10 (10.9)	0	0	0	10 (5.2)
CSF	5 (5.5)	0	3 (5.7)	0	8 (4.1)
Bone	2 (2.2)	0	0	0	2 (1.0)
Other biopsies	7 (7.7)	0	0	1 (33.3)	8 (4.1)
Age, y, mean (± SD)	54.2 (±23.2)	63.7 (±12.4)	63.1 (±15.0)	70 (±0.0)	57.19 (±19.3)
Male	62 (68.1)	34 (73.9)	35 (66.0)	3 (100.0)	131 (67.9)
Host factor, underlying pathology					
None	47 (51.6)	0	0		47 (24.4)
Hematological malignancy	23 (25.3)	24 (52.2)	14 (26.4)		61 (31.6)
Myeloid cell line	8 (34.8)	13 (54.2)	1 (7.1)		22 (36.1)
Lymphoid cell line	15 (65.2)	11 (45.8)	13 (92.8)		39 (63.9)
Stem cell transplant	6 (6.6)	7 (15.2)	1 (1.9)		14 (7.3)
Solid organ transplant	5 (5.5)	6 (13.0)	8 (15.1)	2 (67.7)	21 (10.9)
Lung	1 (20.0)	4 (66.7)	4 (50.0)		9 (42.9)
Liver	2 (40.0)				2 (9.5)
Renal	2 (40.0)	2 (33.3)	4 (50.0)		10 (47.6)
Long-term corticosteroid or immunosuppressant therapy or TKI	0	3 (6.5)	12 (22.6)	1 (33.3)	16 (8.3)
Cancer chemotherapy	8 (8.8)	5 (10.9)	18 (33.9)		31 (16.1)
Respiratory (tracheal, pulmonary, bronchial)	1 (12.5)	4 (80.0)	7 (38.9)		12 (37.5)
Digestive (esophagus, jejunum, rectal, liver)	1 (12.5)		3 (16.7)		5 (16.1)
Renal	2 (25.0)		1 (5.6)		3 (9.7)
Breast/prostate/penis	4 (50.0)		5 (27.8)		9 (29.0)
Epidermoid		1 (20.0)	1 (5.6)		2 (6.5)
Mesothelioma			1 (5.6)		1 (3.2)
Other (severe malnutrition, AIDS, genetic aplasia)	2 (2.2)	1 (2.2)			3 (1.6)
Clinical and/or radiological criteria					
None	71 (78.1)				71 (36.8)
Invasive pulmonary aspergillosis	20 (21.9)	46 (100.0)	51 (96.2)	2 (67.7)	119 (61.7)
Cavity	1 (5.0)	1 (2.2)	8 (15.7)		10 (8.4)
Dense lesions, with or without halo	1 (5.0)	4 (8.7)			5 (4.2)
Nodules or lobar condensation	18 (90.0)	41 (89.1)	43 (84.3)	2 (100.0)	104 (87.4)
Gas crescent					
Cerebral aspergillosis			2 (3.8)	1 (33.3)	3 (1.5)
Mycological criteria (multiple tests possible per patient)					
Total	97	46	86	4	233
None	70 (72.2)	46 (100.0)			116 (49.8)
Isolation of *Aspergillus* species in culture	13 (13.4)		34 (39.5)	1 (33.3)	48 (20.6)
*Aspergillus fumigatus*	10 (76.9)		30 (88.2)	1 (100.0)	41 (85.4)
*Aspergillus* other	2 (15.3)		4 (11.8)		7 (14.6)
Positive Galactomannan	5 (5.2)		26 (30.2)		31 (13.3)
Serum			7 (26.9)		7 (22.6)
BAL	4 (80.0)		15 (57.7)		19 (61.3)
Both BAL and serum			4 (15.4)		4 (12.9)
CSF	1 (20.0)				1 (3.2)
Positive molecular diagnosis for *Aspergillus fumigatus*	9 (9.3)		26 (30.2)	1 (33.3)	36 (15.5)
Plasma	2 (22.2)		2 (8.3)		4 (11.8)
BAL			15 (62.5)		15 (44.1)
Both BAL and plasma					
Other respiratory sample			5 (20.8)		5 (14.7)
Other sample (biopsy, CSF, bone)	7 (77.7)		4 (16.7)	1 (100.0)	5 (14.7)
Histopathological proof				2 (50.0)	2 (0.9)

^*a*^ Variables are expressed as *n* (%) unless otherwise specified.

^*b*^ For each sample, only the main host criterion and the main clinical and/or radiological criterion were retained for the corresponding patient. For mycological criteria, "positive" is defined as meeting the EORTC-MSGERC mycological criteria (7). Each sample could be considered positive by multiple mycological tests. Other respiratory sample includes bronchial aspirate, tracheal aspirate, and sputum.

^*c*^ EORTC-MSGERC, European Organisation for Research and Treatment of Cancer and Mycoses Study Group Education and Research Consortium; IA, invasive aspergillosis; TKI, tyrosine kinase inhibitor; AIDS, acquired immunodeficiency syndrome; BAL, bronchoalveolar fluid; CSF; cerebrospinal fluid.

### Sensitivity and specificity comparison between RT-qPCR and qPCR

When considering all sample types, RT-qPCR was significantly more sensitive than qPCR for all classifications (possible, probable, proven) and the probable IA only modalities, with an additional 17/102 and 7/53 samples detected by RT-qPCR, respectively (*P*-value < 0.01 and <0.05, respectively). For plasma samples only, sensitivities were significantly higher and specificities significantly lower using RT-qPCR for the three classification modalities (*P*-value < 0.001, <0.05 and <0.05 for all classifications, probable IA only, and probable and proven IA, respectively). For respiratory samples, there was no significant difference in sensitivity or specificity between the RT-qPCR and qPCR (Table 2).

**TABLE 2:** Analytical performance of the RT-qPCR and qPCR for the diagnosis of invasive aspergillosis according to clinical classification and sample type*^a^*^,^*^b^*

		All classifications (possible, probable, proven)		Probable		Probable and proven	
		RT-qPCR	qPCR	RT-qPCR	qPCR	RT-qPCR	qPCR
All samples	*Se*	0.66 ** [0.56–0.74]	0.49 [0.39–0.58]	0.87 * [0.75–0.93]	0.74 [0.60–0.84]	0.86 [0.74–0.93]	0.73 [0.60–0.83]
	*Sp*	0.79 [0.70–0.86]	0.91 * [0.83–0.95]	0.79 [0.69–0.86]	0.91 * [0.84–0.96]	0.79 [0.69–0.86]	0.91 * [0.84–0.95]
Plasma samples	*Se*	0.34 *** [0.19–0.53]	0.00 [0.0–0.12]	0.43 * [0.16–0.75]	0.00 [0.0–0.35]	0.38 * [0.14–0.69]	0.00 [0.0–0.32]
	*Sp*	0.81 [0.63–0.92]	1.0 ** [0.88–1.0]	0.81 [0.63–0.92]	1.00 ** [0.88–1.00]	0.81 [0.63–0.92]	1.00 ** [0.88–1.00]
Respiratory samples^*c*^	*Se*	0.78 [0.67–0.86]	0.71 [0.59–0.80]	0.95 [0.85–0.99]	0.91 [0.78–0.96]	0.95 [0.85–0.99]	0.91 [0.79–0.96]
	*Sp*	0.85 [0.68–0.91]	0.90 [0.77–0.96]	0.80 [0.66–0.89]	0.90 [0.77–0.96]	0.83 [0.68–0.91]	0.90 [0.77–0.96]

^*a*^ Sensitivity (Se) and Specificity (Sp) are provided with their 95% confidence intervals [95% CI].

^*b*^ *: significantly superior with *: *P* < 0.05, **: *P* < 0.01, ***: *P* < 0.001.

^*c*^ Respiratory sample includes bronchoalveolar fluid, bronchial aspirate, tracheal aspirate, and sputum.

### Agreement values between RT-qPCR and qPCR

For all sample types, overall agreement rates between RT-qPCR and qPCR were 85.5%, 87.5%, and 87.8% for all classifications, probable IA, and probable and proven IA, respectively, while Kappa coefficients presented substantial agreement for all sample modalities. For plasma samples, overall agreement rates were 73.2%, 76.5%, and 77.1% for all classifications, probable IA, and probable and proven IA, respectively, while Kappa coefficients were not calculable as there were no positive qPCR results. For respiratory samples, overall agreement rates were 92.7%, 94.0%, and 94.0% for all classifications, probable IA, and probable and proven IA, respectively, while Kappa coefficients presented an almost perfect agreement for all sample modalities (Table 3).

**TABLE 3:** Agreement values and kappa coefficient between qPCR and RT-qPCR according to clinical classification and sample type^*a*^^,^^*b*^

	All classifications (possible, probable, proven)	Probable	Probable and proven
All samples			
Overall agreement %	85.5 [79.8–89.8]	87.5 [81.1–91.9]	87.8 [81.5–92.1]
Positive agreement %	100.0 [93.8–100.0]	100.0 [92.4–100.0]	100.0 [95.4–100.0]
Negative agreement %	79.3 [71.7–85.2]	81.4 [72.6–87.9]	73.1 [61.5–82.3]
Kappa coefficient	0.69 [0.59–0.79]	0.75 [0.64–0.85]	0.75 [0.64–0.85]
Kappa interpretation	Substantial agreement	Substantial agreement	Substantial agreement
Plasma samples			
Overall agreement %	73.2 [60.4–83.0]	76.5 [60.0–87.6]	77.1 [61.0–87.9]
Positive agreement %	NA	NA	NA
Negative agreement %	73.2 [60.4–83.0]	76.5 [60.0–87.6]	77.1 [61.0–87.9]
Kappa coefficient	NA	NA	NA
Kappa interpretation	NA	NA	NA
Respiratory samples			
Overall agreement %	92.7 [86.2–96.2]	94.0 [86.7–97.4]	94.0 [86.8–97.4]
Positive agreement %	100.0 [93.2–100.0]	100.0 [91.8–100.0]	100.0 [92.0–100.0]
Negative agreement %	85.7 [74.3–92.6]	87.5 [73.9–94.5]	87.5 [73.9–94.5]
Kappa coefficient	0.85 [0.76–0.95]	0.88 [0.78–0.98]	0.88 [0.78–0.98]
Kappa interpretation	Almost perfect agreement	Almost perfect agreement	Almost perfect agreement

^*a*^ For agreement calculation, qPCR method was considered the comparative method and RT-qPCR as the candidate method. Agreements and Kappa coefficient are provided with their 95% confidence intervals [95% CI].

^*b*^ NA, not applicable.

### Ct comparison between RT-qPCR and qPCR

**Fig 1:**
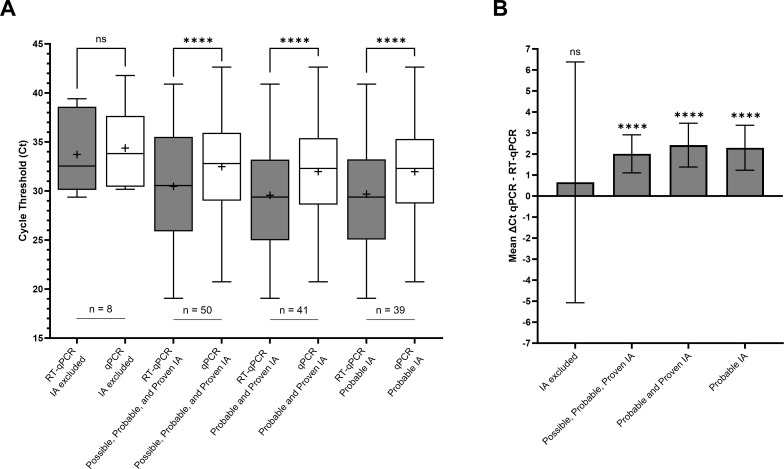
(**A**) Comparison of mean cycle thresholds between RT-qPCR and qPCR according to different invasive aspergillosis classification modalities. (**B**) Corresponding mean ΔCt, computed as the difference between the Ct obtained in qPCR and that obtained in RT-qPCR. IA classification was performed using the EORTC-MSGERC criteria. In Fig. 1A, the middle line of the boxplot indicates the median, and the cross indicates the mean. The bar indicates the minimum and maximum values. In Fig. 1B, the bar indicates the CI95% of the mean ΔCt. ns, not significant, ****: *P* < 0.0001. Abbreviation: EORTC-MSGERC, European Organisation for Research and Treatment of Cancer and Mycoses Study Group Education and Research Consortium; IA, invasive aspergillosis.

There was no significant difference in mean Ct between RT-qPCR and qPCR for the IA excluded. The mean Ct obtained with RT-qPCR were significantly lower than those obtained with qPCR for the following modalities: all classifications, probable and proven IA together, and probable IA only (*P*-value < 0.0001 for all three groups; Fig. 1A). For the all-classifications modality, the mean Ct were 30.47 [95% CI 28.81–32.13] and 32.48 [95% CI 31.11–33.85] for RT-qPCR and qPCR, respectively, corresponding to a mean ΔCt of 2.01 [95% CI 1.10–2.91]. For the probable and proven IA modality, the mean Ct were 29.56 [95% CI 27.72–31.40] and 31.98 [95% CI 30.41–33.55] for RT-qPCR and qPCR, respectively, corresponding to a mean ΔCt of 2.42 [95% CI 1.38–3.46]. For the probable IA alone modality, the mean Ct were 29.68 [95% CI 27.80–31.55] and 31.98 [95% CI 30.34–33.61] for RT-qPCR and qPCR, respectively, corresponding to a mean ΔCt of 2.29 [95% CI 1.23–3.37] (Fig. 1B).

**Fig 2:**
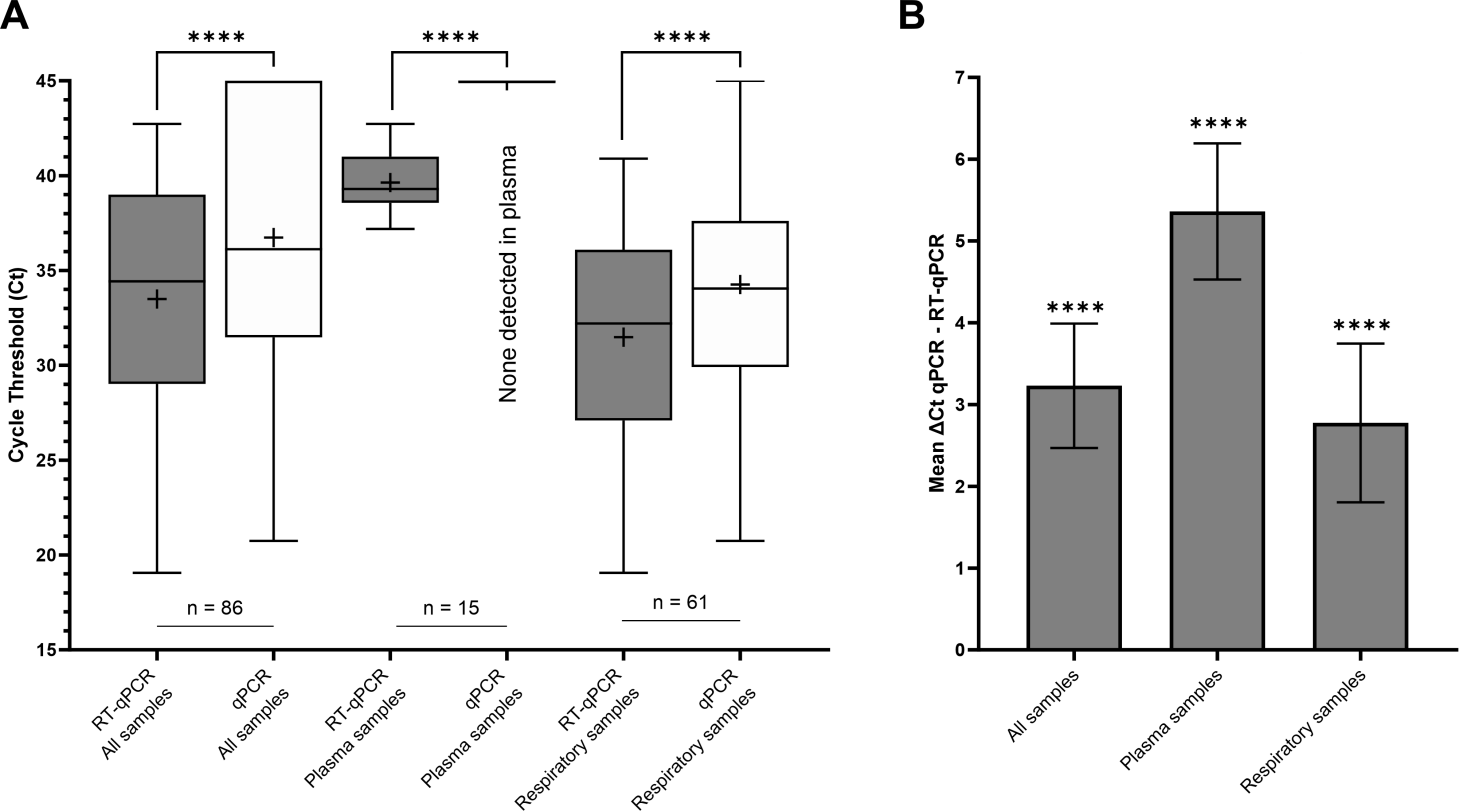
(**A**) Comparison of mean cycle thresholds between RT-qPCR and qPCR according to different sample types. (**B**) Corresponding mean ΔCt, computed as the difference between the Ct obtained in qPCR and that obtained in RT-qPCR. In Fig. 1A, the middle line of the boxplot indicates the median, and the cross indicates the mean. The bar indicates the minimum and maximum values. In Fig. 1B, the bar indicates the CI95% of the mean ΔCt. ****: *P* < 0.0001.

The mean Ct obtained with RT-qPCR were significantly lower than those obtained with qPCR for all modalities: all samples, plasma samples, and respiratory samples (*P* < 0.0001; Fig. 2A). For all samples, the mean Ct were 33.50 [95% CI 32.17–34.83] and 36.73 [95% CI 35.24–38.23] for RT-qPCR and qPCR, respectively, corresponding to a mean ΔCt of 3.23 [95% CI 2.47–3.99]. For plasma samples, the mean Ct for RT-qPCR was 39.64 [95% CI 38.81–40.47], and all qPCR results were negative; the mean ΔCt was 5.36 [95% CI 4.53–6.19]. For respiratory samples, the mean Ct were 31.48 [95% CI 30.02–32.95] and 34.26 [95% CI 32.70–35.82] for RT-qPCR and qPCR, respectively, corresponding to a mean ΔCt of 2.78 [95%CI 1.80–3.74] (Fig. 2B).

## DISCUSSION

The results of the present study suggest that RT-qPCR represents an advance in IA diagnosis over conventional qPCR methods.

The present findings show that the sensitivity of the RT-qPCR was higher than that of the qPCR. This was particularly the case for plasma samples, enabling the detection of *Aspergillus fumigatus* genetic material in samples with very low fungal loads. Indeed, the Ct values obtained with RT-qPCR exceeded 40 in almost half of the plasma samples tested, explaining the fact that the qPCR did not detect *Aspergillus fumigatus* in any of these samples. Since it is extremely rare to obtain a positive culture of *Aspergillus* from blood, whatever the sample type (plasma, serum, or blood cultures) (29), increasing the ability to detect low levels of cell-free TNA in circulating blood using RT-qPCR would allow the use of non-invasive samples for the diagnosis of IA. Another test routinely used for the diagnosis of IA from blood samples is the detection of GM, the values of which have been shown to correlate well with the amount of DNA detected (27). The latter study also showed that molecular testing can detect *Aspergillus* DNA earlier than GM although more transiently and at low levels. The combined use of GM detection and RT-qPCR could, therefore, increase the diagnostic performance of IA in blood samples. This is of particular interest since the use of blood samples for molecular detection enables a faster management of the patient and is less invasive than BAL sampling, which involves transferring patients to an endoscopy department, local anesthesia, and possible adverse effects in patients with comorbidities. Moreover, using blood offers the possibility for repeated analyses, which contributes to improving sensitivity and eliminating suspicions of contamination at the collection stage. Besides, improving the sensitivity for detecting *Aspergillus fumigatus* would also be of interest in other sample types with low fungal load and low culture sensitivity, such as CSF (19). In such samples containing small quantities of detectable nucleic acid, the process of nucleic acid extraction from the sample constitutes a critical step; optimizing this step could significantly improve the performance of subsequent PCRs (30).

The increased sensitivity of a molecular testing technique may be associated with a reduced specificity, as observed herein for all sample types. This is particularly relevant when dealing with airborne fungi which are ubiquitous in the environment, as is the case of *Aspergillus fumigatus*. This loss of specificity can be partially overcome by establishing Ct thresholds specific to each sample type, enabling a distinction between colonization and infection status. Such thresholds, however, are difficult to establish due to the non-standardization of sampling protocols for certain sample types, such as BAL, which can lead to variations in the quantity of fungal material sampled, thus altering analytical performances. Importantly, the diagnosis of IA is not based solely on the molecular testing result, but on a combination of clinical, radiological, and other mycological analyses. By combining conventional mycological analyses, antigenic detection, and molecular testing, the diagnosis of IA can be achieved with a sensitivity and specificity of at least 90% (31). Therefore, the lower specificity observed herein using the RT-qPCR should have a limited clinical impact, since the decision to introduce antifungal treatment is based on multiple considerations. In our cohort, only six patients had iterative plasma sampling, and both RT-qPCR and qPCR assays returned negative for all samples from five patients, only two samples from the sixth patient returned positive by RT-qPCR assay, making it difficult to assess the benefit of iterative blood sampling for the diagnosis of IA with these limited data; for the other positive RT-qPCR assays performed in plasma, there was no further testing because either the GM test or the culture of the associated sample was positive. Furthermore, this does not allow us to discuss the usefulness in “real life” of the requirement for two positive PCRs in blood to satisfy the mycological criterion. Analysis of a larger cohort selected directly on the presence of a blood sample for a molecular biology test for AI could enable us to evaluate this endpoint.

The present study has several limitations related to the number of samples included and its retrospective design. Since only samples on which the realization of a molecular diagnostic test was performed were included, this limited the number of certain sample types available. For instance, in our center, the diagnosis of IA on blood samples is often carried out by GM rather than molecular testing explaining the relatively low number of plasma samples included over the 2-year period. Similarly, in “precious" samples, such as biopsies and CSF, culture is generally preferred because a positive result enables an AST to be performed; the low number of such samples, thus, limited the performance analysis of the RT-qPCR method for rare forms of IA. Setting up a prospective study using systematic molecular testing for these types of samples would, thus, be essential, but it could be a long and costly process considering the rarity of these diseases.

### Conclusion

The present study demonstrated the higher sensitivity of RT-qPCR over qPCR for the diagnosis of invasive aspergillosis due to *Aspergillus fumigatus*, particularly in samples with an intrinsically low fungal load. These findings strengthen the usefulness of having included molecular testing in the mycological criteria and call for the development of innovative molecular techniques for the diagnosis of invasive fungal diseases.

## SUPPLEMENTAL MATERIAL

Table S1 jcm.00791-24-s0001.docx Contingency table of RT-qPCR and qPCR results according to EORTC-MSGERC clinical classification and sample type.

ASM does not own the copyrights to Supplemental Material that may be linked to, or accessed through, an article. The authors have granted ASM a non-exclusive, world-wide license to publish the Supplemental Material files. Please contact the corresponding author directly for reuse.
