# Supplementary material for: Reverse-transcriptase real-time PCR in the diagnostic strategy for invasive infections caused by Aspergillus fumigatus
Source: J Clin Microbiol. 2024 Oct 24;62(11):e00791-24. doi: 10.1128/jcm.00791-24 (PMC11559004; doi:10.1128/jcm.00791-24)
Supplement: Table S1 — Contingency table of RT-qPCR and qPCR results according to EORTC-MSGERC clinical classification and sample type. [file jcm.00791-24-s0001.docx]

Supplemental table 1. Contingency table of RT-qPCR and qPCR results according to EORTC-MSGERC clinical classification and sample type.

|  | All classifications  (Possible, Probable, Proven) | | Probable | | Probable and Proven | |
| --- | --- | --- | --- | --- | --- | --- |
|  | IA | No IA | IA | No IA | IA | No IA |
| All samples | | | | | | |
| RT-qPCR |  |  |  |  |  |  |
| Positive | 67 | 19 | 46 | 19 | 48 | 19 |
| Negative | 35 | 72 | 7 | 72 | 8 | 72 |
| qPCR |  |  |  |  |  |  |
| Positive | 50 | 8 | 39 | 8 | 41 | 8 |
| Negative | 52 | 83 | 14 | 83 | 15 | 83 |
| Total | 102 | 91 | 53 | 91 | 56 | 91 |
| Plasma samples | | | | | | |
| RT-qPCR |  |  |  |  |  |  |
| Positive | 10 | 5 | 3 | 5 | 3 | 5 |
| Negative | 19 | 22 | 4 | 22 | 5 | 22 |
| qPCR |  |  |  |  |  |  |
| Positive | 0 | 0 | 0 | 0 | 0 | 0 |
| Negative | 29 | 27 | 7 | 27 | 8 | 27 |
| Total | 29 | 27 | 7 | 27 | 8 | 27 |
| Respiratory samples | | | | | | |
| RT-qPCR |  |  |  |  |  |  |
| Positive | 54 | 7 | 41 | 7 | 42 | 7 |
| Negative | 15 | 33 | 2 | 33 | 2 | 33 |
| qPCR |  |  |  |  |  |  |
| Positive | 49 | 4 | 39 | 4 | 40 | 4 |
| Negative | 20 | 36 | 4 | 36 | 4 | 36 |
| Total | 69 | 40 | 43 | 40 | 44 | 40 |
